# Separating Vegetation Greening and Climate Change Controls on Evapotranspiration trend over the Loess Plateau

**DOI:** 10.1038/s41598-017-08477-x

**Published:** 2017-08-15

**Authors:** Zhao Jin, Wei Liang, Yuting Yang, Weibin Zhang, Jianwu Yan, Xuejuan Chen, Sha Li, Xingguo Mo

**Affiliations:** 1 School of Geography and Tourism, Shaanxi Normal University, Xi’an, 710119 China; 2 National Demonstration Center for Experimental Geography Education, Shaanxi Normal University, Xi’an, 710119 China; 3 State Key Laboratory of Urban and Regional Ecology, Research Center for Eco-Environmental Sciences, Chinese Academy of Sciences, Beijing, 100085 China; 4 CSIRO Land and Water, Canberra, ACT 2601 Australia; 5 Key Laboratory of Water Cycle and Related Land Surface Processes, Institute of Geographical Sciences and Natural Resources Research, Chinese Academy of Sciences, Beijing, 100101 China

## Abstract

Evapotranspiration (ET) is a key ecological process connecting the soil-vegetation-atmosphere system, and its changes seriously affects the regional distribution of available water resources, especially in the arid and semiarid regions. With the Grain-for-Green project implemented in the Loess Plateau (LP) since 1999, water and heat distribution across the region have experienced great changes. Here, we investigate the changes and associated driving forces of ET in the LP from 2000 to 2012 using a remote sensing-based evapotranspiration model. Results show that annual ET significantly increased by 3.4 mm per year (*p* = 0.05) with large interannual fluctuations during the study period. This trend is higher than coincident increases in precipitation (2.0 mm yr^−2^), implying a possible pressure of water availability. The correlation analysis showed that vegetation change is the major controlling factor on interannual variability of annual ET with ~52.8% of pixels scattered in the strip region from the northeastern to southwestern parts of the LP. Further factorial analysis suggested that vegetation greening is the primary driver of the rises of ET over the study period relative to climate change. Our study can provide an improved understanding of the effects of vegetation and climate change on terrestrial ecosystem ET in the LP.

## Introduction

As a nexus of water, energy and carbon cycles, land evapotranspiration (ET) is a critical process for the climate system and biogeochemical cycles in terrestrial ecosystems^1^. Global land ET gives back about 60% of annual terrestrial precipitation to the atmosphere^2–4^. Changes in ET and relevant latent heat flux can, on one hand, help to control land surface temperature which significantly reflects regional and global climate characteristics such as the intensity and duration of heat waves^5^. On the other hand, changes in ET can influence land surface water availability in water bodies such as lakes and rivers^4^. Therefore, understanding of magnitudes, mechanisms and interactions that control historical ET dynamics is a prerequisite for predicting future changes in ET patterns^6^, but also are crucial to solve a wide range of problems in hydrology, geographical ecology and water resource management, especially in the arid and semiarid regions^7, 8^.

Recent researches have indicated that hydrological cycling has accelerated with climate change and anthropogenic effects, which would alter global land surface processes through ET^9, 10^. ET is controlled by the combination of atmospheric evaporative demand (potential evapotranspiration), the energy available at the surface (solar radiation), and water supply^11, 12^. Therefore, it is a large yet diverse part of the water budget and extraordinary heterogeneity in space-time^13^. Some studies have been carried out on the ET changes and associated driving forces in the last several decades, but there are significant differences between different regions. For example, although vegetation greening and climate change promoted multidecadal rises of global land ET^12^, increasing soil moisture limitation lead to the recent ET decline from 1998 to 2008^1^. Guo and Shen^14^ found that ET has generally decreased during the period from 1981 to 2008 in the plain regions in Haihe River Basin, North China, which could be attributed to less net radiation and precipitation as well as rapid urbanization. Eslamian, *et al*.^15^ believed that rising air temperature and declining relative humidity could enhance the evaporative capability and decrease the actual vapor, which, in turn, caused the increase of ET in arid and semi-arid regions of Iran from 1951 to 2005. Other studies have also found that changes in meteorological variables may have a combined influence on the potential evapotranspiration of the atmosphere, and thus ET in the Yellow River Basin of China over the past 50 years^16^.

Numerous methods have been used to quantify ET. *In situ* measurements, such as weighing lysimeter, energy balance Bowen ratio systems, and eddy covariance technique are generally believed to be reliable methods for ET estimates^17^. However, these methods are limited to measurement of a small scale (point scale, field scale, landscape scale) and therefore they are not applicable in obtaining details of ET datasets for spatial distribution in terms of a long time series. Similarly, traditional ET estimation models, whose parameters derived mostly from field observations, are greatly restricted to be used at a small scale, i.e., soil-plant-atmosphere continuum models^8^. This hinders a further understanding of the change rules in the process of regional ET.

Satellite remote sensing, fortunately, which is able to capture land surface information from larger geographic extents, thus providing an effective tool and method for deriving the ground parameters for ET simulation at the large scale^8^. By integrating remote sensing vegetation index with ground-based climate data, numerous process-based models have been used to meet the requirement of evaluating large-scale ET^14, 18^, such as Two-Source Energy Balance (TSEB)^19^, the Surface Energy Balance System (SEBS)^20^, the Hybrid dual-source Trapezoid Evapotranspiration Model (HTEM)^21^, the Simplified Surface Energy Balances Index (S-SEBI)^22^, and the Surface Energy Balances Algorithm for Land (SEBAL)^23^. Nevertheless, there is still a need for modeling studies, since the restrictions of complex mechanisms, multitudinous parameters and requiring uniform land surface have made most previous models uncertainties when used in the area with complex topography and geology. Herein, we use the remote sensing model, the Vegetation Index model^24–29^, which integrates the products of remote sensing vegetation index with fewer parameters, to estimate ET because it can better reflect the macroscopical pattern of a region.

The Loess Plateau (LP), the main portion of the Yellow River, is an ecologically vulnerable region and is well known for its high soil erosion rates, contributing ~90% of sediments in the Yellow River^30^. In order to prevent soil erosion and restore the ecological functions of watersheds, the Chinese government has launched the Grain-for-Green (GFG) project, which is the largest active revegetation project in China or anywhere else^31^. This project has greatly improved vegetation growth, and it was reported that the total afforested area of Ningxia, Shaanxi, and Shanxi during the period from 1999 to 2012 have reached 3.8 × 10^4^ km^2^, accounting for 11.2% of the land area of the three provinces^32^. However, changes of vegetation cover will affect underlying surface characteristics and have both direct and indirect impacts on the local surface water and energy budget^33^. Over the past few decades, concerns have been arisen with arguments that large-scale vegetation restorations may result in water shortage^34, 35^. Comparatively, ET is a better time integrator of regional change than precipitation and streamflow, since the latter two variables are intermittent and non-linear processes whereas ET occurs every day^36^. Consequently, quantitative estimation of ET of the LP and further findings in the main driving factors for ET changes can significantly improve the eco-environment, balance the water consumption and properly allocate available water resources.

There are some studies on the effect of land use/land cover changes on ET. Zhang, *et al*.^37^ suggested that the characteristics of land use/cover in the LP basically control the regional distribution characteristics of ET. Li, *et al*.^38^ thought that the conversion from shrub land and sparse woodland to medium and high coverage grassland resulted in increased ET in an agricultural catchment on the LP from 1981 to 2000. However, with the expansion of the “green area” in the plateau, the regional ET is showing a significant change with a high degree of uncertainty, and few studies have focused on the changes and associated driving forces of ET in the LP in terms of human intervention (i.e., GFG project). To bridge this gap, we examined vegetation greening and climate change controls on ET over the Loess Plateau for the period 2000–2012 using the vegetation index model.

## Results

### Validation of the model

**Figure 1:**
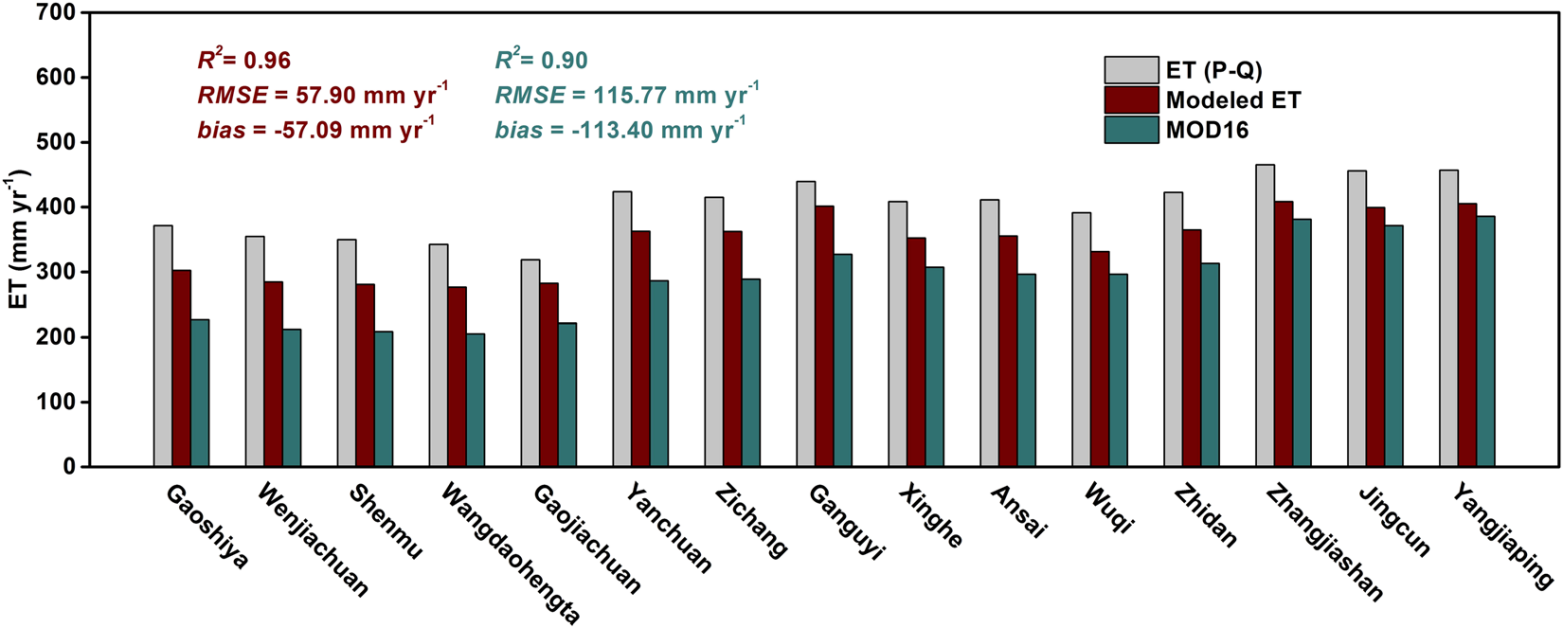
Validation of the modeled evapotranspiration (ET). Comparison of the estimated annual mean ET (2000–2011) and MODIS ET to observed ET (precipitation minus streamflow) in 15 catchments on the Loess Plateau (LP).

Over the long-term period, the comparisons between simulated ET and observed mean ET (precipitation minus streamflow) for the 15 catchments from 2000 to 2011 indicated that the model generally performed well as judged by model statistics (Fig. 1, *R* ^2^ = 0.96; RMSE = 57.90 mm yr^−1^). The mean bias (bias, defined as modeled mean minus observed mean) of the estimated ET was −57.09 mm yr^−1^, suggesting that the model slightly underestimated ET in these catchments. This result is reasonable because the model failed to consider the effect of water evaporation from the check-dams and irrigation.

Compared with validation of the MODIS ET products, we can know clearly that the MODIS ET products in the region underestimated much more, with *R* ^2^ = 0.90, RMSE = 115.77 mm yr^−1^ and bias = −113.40 mm yr^−1^ (Fig. 1). In other words, the simulated ET values were closer to the observed values. Generally speaking, the good performance, as indicated by relatively high *R* ^2^ and low RMSE and bias, indicates that the model has a good potential to be used for analyzing ET patterns in the LP.

### Spatial pattern of ET, P, PET and LAI

**Figure 2:**
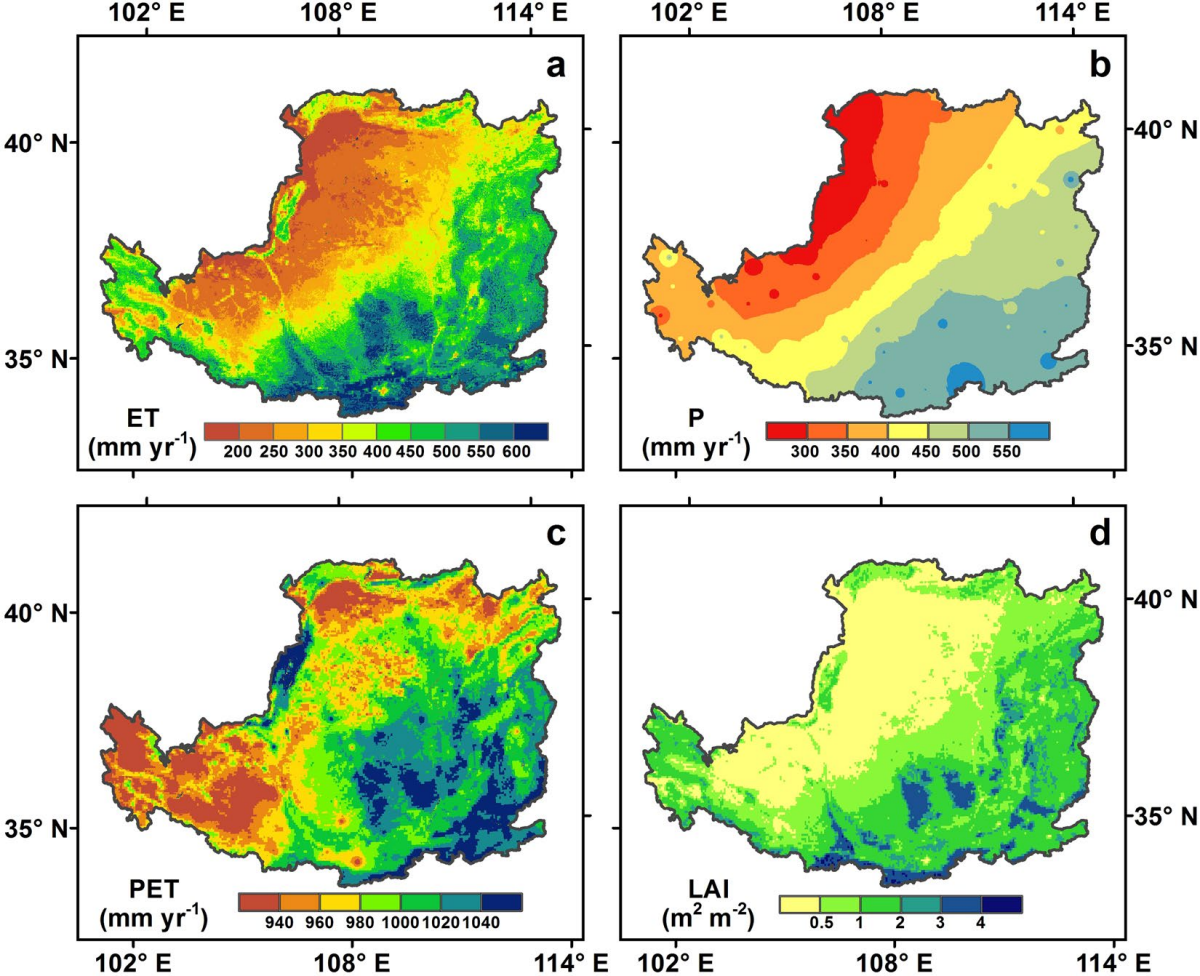
Spatial patterns of mean annual (**a**) ET, (**b**) precipitation (P), (**c**) potential evapotranspiration (PET), and (**d**) growing season leaf area index (LAI) from 2000 to 2012 on the LP. Map was created using ArcMap 10.0 (http://www.esri.com/sofware/arcgis/arcgis-for-desktop).

Averaged spatially over the whole region, the mean annual ET was 379 ± 22 mm yr^−1^ over the last thirteen years, which accounted for 90% of the corresponding precipitation (P), suggesting that most of precipitation gives back to the atmosphere through evaporation. The spatial pattern of mean annual ET over the LP from 2000 to 2012 was shown in Fig. 2a. Overall, the ET from the model has exhibited large spatial variability across the regions, decreasing gradually from the southeastern to northwestern areas, which is consistent with that of annual P and LAI in the growing season (monthly average from April to October) (Fig. 2b and d). Annual ET was generally higher than 400 mm yr^−1^ in extensive the southeastern LP, where croplands and productive forests were densely distributed (Fig. S1). Meanwhile, these areas were also characterized by a warm temperature and relatively abundant P, and thus were suitable for vegetation growth. In comparison, annual ET less than 200 mm yr^−1^ usually corresponds to grass and desert (Fig. S1), and climate was featured by local low P (<200 mm yr^−1^). As shown in Fig. 2b, the mean annual P was 420 ± 50 mm yr^−1^, and increased from 300 mm yr^−1^ to 560 mm yr^−1^ from the northwest to southeast of the LP.

The mean annual potential evapotranspiration (PET) was 988 ± 26 mm yr^−1^ and the regional difference ranged from 900 to 1070 mm yr^−1^ over the LP from 2000 to 2012 (Fig. 2c). The lowest PET were found in the north and southwest regions of the LP, which were less than 940 mm yr^−1^, and the highest values (>1040 mm yr^−1^) were distributed in the southeastern, northwest and central parts of the LP, which was consistent with previous studies^38^, By contrast, PET in most areas was more than 920 mm yr^−1^ and in most of the southeastern LP is more than 980 mm yr^−1^, which suggests that the spatial variation of PET on the LP is not prominent.

The relatively high annual LAI (>1 m^2^ m^−2^) was distributed in the most southeastern areas and some parts in the southwestern areas (Fig. 2d); for the remaining areas in northwestern LP, mean annual LAI was blow 0.5 m^2^ m^−2^.

**Figure 3:**
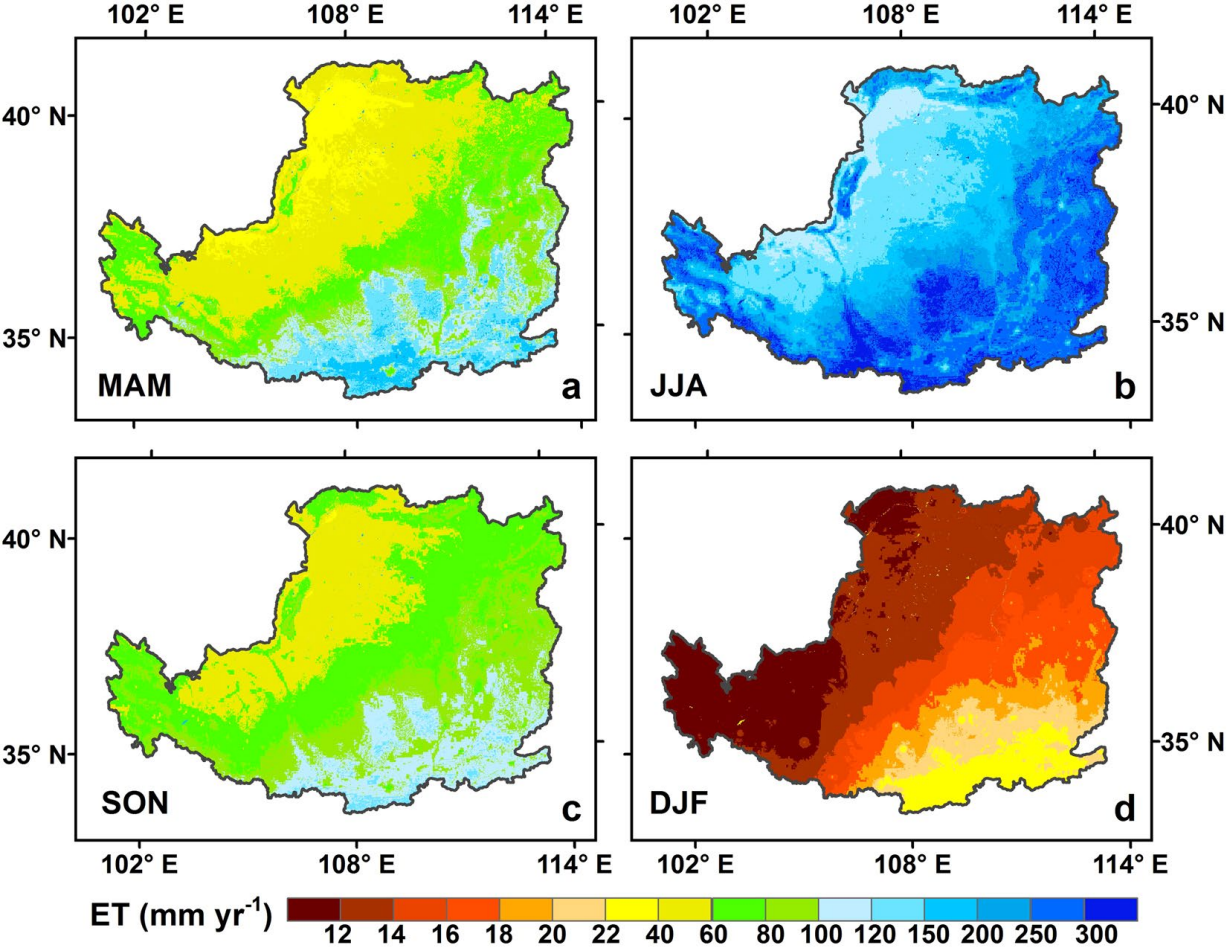
Spatial patterns of mean annual seasonal ET of the LP: (**a**) March, April, and May (MAM), (**b**) June, July, and August (JJA), (**c**) June, July, and August (JJA), and (**d**) December, January and February (DJF). Map was created using ArcMap 10.0 (http://www.esri.com/sofware/arcgis/arcgis-for-desktop).

The spatial patterns of seasonal ET, especially for the summer (June, July and August, JJA) ET, were similar to that of the annual ET, but with different magnitudes (Fig. 3). The magnitude of ET showed gradients decreasing from the northwest to the southeast in all four seasons. Meanwhile, obvious seasonal cycles of ET could be detected. Specifically, during the summer season, mean monthly ET was 209 ± 12 mm yr^−1^, accounting for over 55.3% of the annual ET, with a maximum of 390 mm yr^−1^ and a minimum of 70 mm yr^−1^. In contrast, during spring (March, April and May, MAM) and autumn (September, October and November, SON), monthly ET were relatively smaller because of the unfavorable condition for growth (lower temperature and precipitation). During winter (December, January and February, DJF), ET was less than 30 mm yr^−1^ over most of the region, reflecting vegetation dormancy and low solar energy.

### Trend in ET, P, PET and LAI

**Figure 4:**
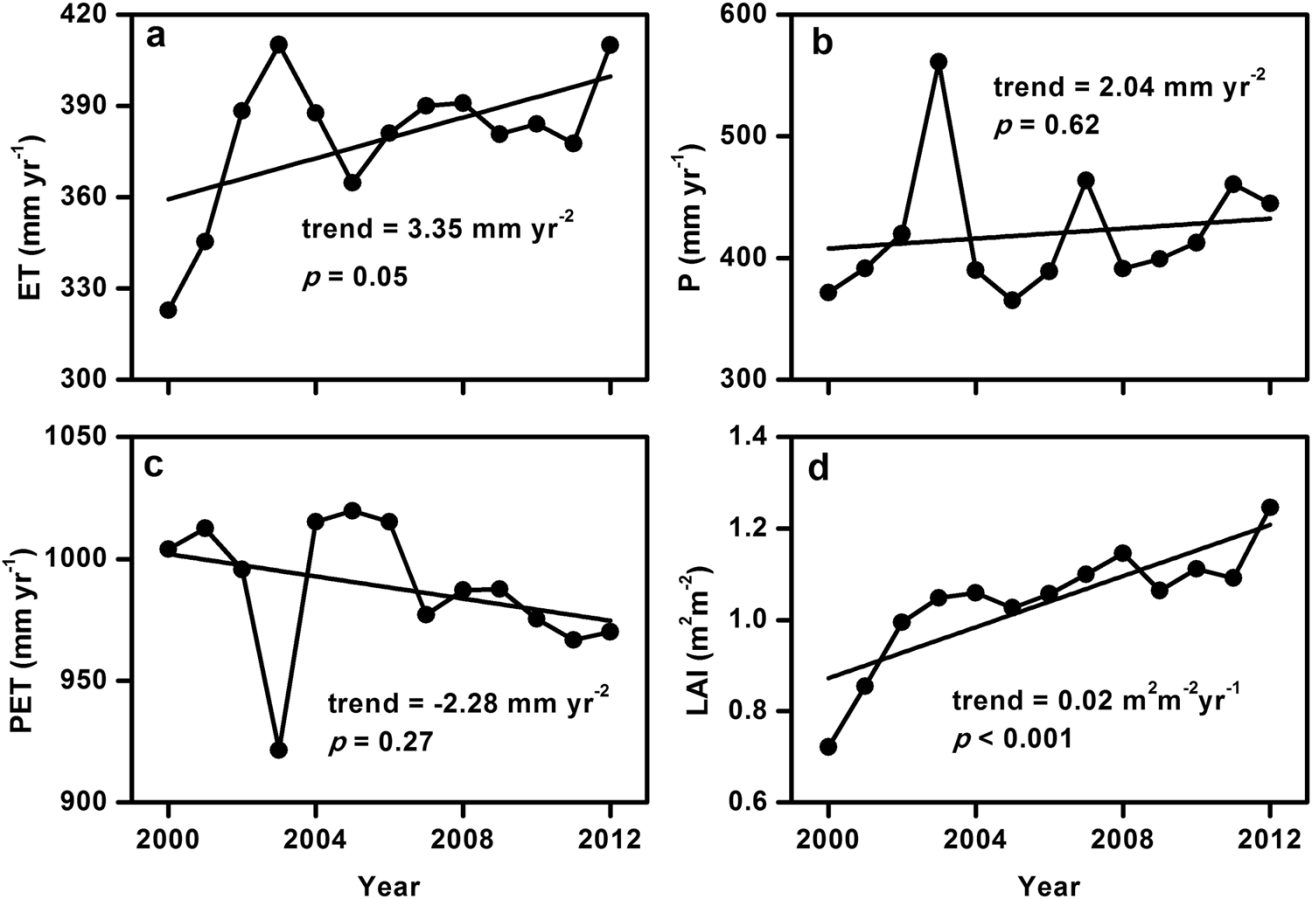
Interannual variations in mean (**a**) ET, (**b**) P, (**c**) PET and (**d**) LAI over the LP from 2000 to 2012 (black dots). Solid black lines show the best fit linear relationship for the whole period.

The interannual variation in ET over the LP from 2000 to 2012 is shown in Fig. 4a. On average, annual ET in the LP significantly increased (*p* < 0.1), with an annual increase of 3.35 mm per year. However, the change of ET was not continuous throughout the 13-year period. The ET experienced a rapidly increasing trend from 2000 to 2003 (*p* < 0.001), and then a significantly decreasing trend occurred in the next 3 years (*p* < 0.001). Such changes in ET corresponded well with that in P which reached the peak in 2003 and followed by a sharp decline to the average level (Fig. 4b). After 2005, the annual ET increased steadily, with an annual increase rate of only 1.98 mm yr^−2^ (*p* = 0.38). Annual P presented an insignificantly increasing trend during the whole study period (*p* = 0.62), with an annual rate of 2.04 mm yr^−2^, lower than that of ET, implying a possible pressure of water availability. Contrary to P, the annual PET decreased with a linear slope of −2.28 mm yr^−2^ (*p* = 0.27), and the lowest value of 921.5 mm occurred in 2003 corresponding to the highest annual P (Fig. 4c). In comparison with meteorological variables, vegetation variable (LAI) showed a significantly increasing trend with less fluctuation, partly due to the vegetation physiological activity adaptation to climate change^39^. The variation of LAI were in good agreement with the annual variation of ET at each of the three periods, especially for the period of 2006–2012. The greening trend estimated from the satellite growing season (April to October) LAI was 0.02 m^2^ m^−2^ yr^−1^ (Fig. 4d), lower than that of the global average level (0.068 ± 0.045 m^2^ m^−2^ yr^−1^)^40^.

**Figure 5:**
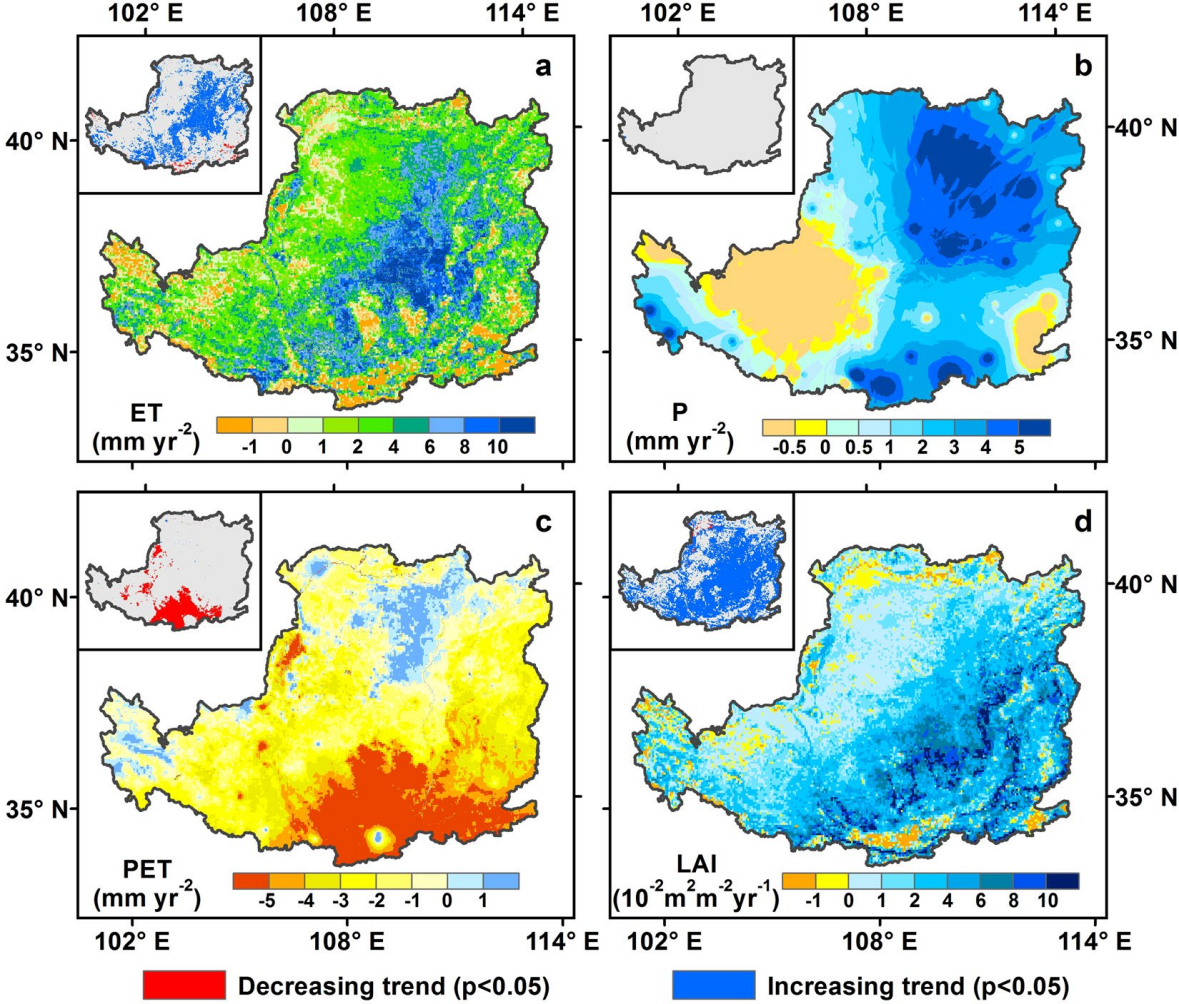
Spatial distributions of annual (**a**) ET, (**b**) P, (**c**) PET, and (**d**) LAI trends in the LP from 2000 to 2012. Map was created using ArcMap 10.0 (http://www.esri.com/sofware/arcgis/arcgis-for-desktop).

As shown in Fig. 5, the spatial distribution patterns of trends in annual ET, P, PET and growing seasonal LAI in the LP presented prominent geographical heterogeneity. During the period 2000–2012, the annual ET increased ~86.6% and significantly increased ~28.0% (*p* < 0.05) in the study area. The highest trend occurred in the strip from the northeastern to the southwestern of the LP. By contrast, only about 13.3% (1.5%) of the study areas experienced a (significantly) decreasing ET trend, which were scattered in the southeast and northwest of the LP. In comparison to ET, temporal changes of annual P showed a mixed pattern of upward and downward trend in the LP (Fig. 5b). The annual P at ~78.6% of the study areas experienced an increasing trend, with the highest value (>4 mm yr^−2^) occurred in the northern and southern regions. However, only ~0.04% (*p* < 0.05) significantly increased. Conversely, annual P at only ~21.4% of the study areas experienced a slightly decreasing trend which was mainly distributed in the western and eastern LP. Compared with P, the annual PET decreased over large areas in the LP (87.3%) (Fig. 5c), with the sharpest decrease in the south part of the LP. Regions with increased annual PET were mainly located in the central of the northern LP. For LAI, it was shown that the greening trends were overwhelming (91.4%), and the most prominent greening trends were distributed in the strip area between the northeastern and southeastern of the study region (Fig. 5d).

**Figure 6:**
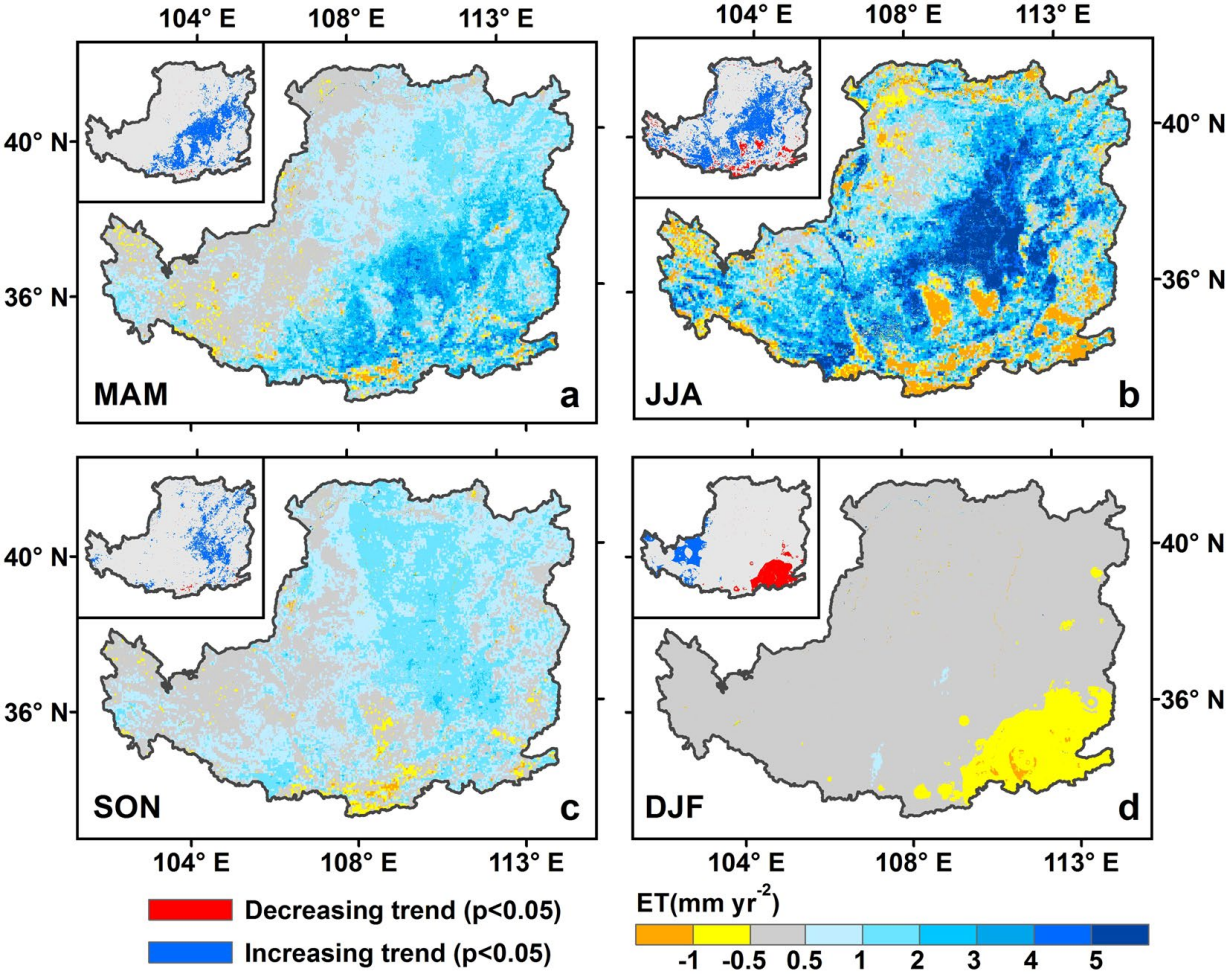
Spatial distributions of seasonal ET trend in the LP from 2000 to 2012: (**a**) MAM, (**b**) JJA, (**c**) SON, and (**d**) DJF. Map was created using ArcMap 10.0 (http://www.esri.com/sofware/arcgis/arcgis-for-desktop).

The spatial distributions of seasonal ET trends during the period 2000–2012 on the LP are depicted in Fig. 6. There were obvious differences in spatial distribution trends patterns of ET for different seasons. ET in other seasons experienced an increasing tendency except in winter (DJF), with the trend of summer (JJA) being the closest to that of the annual ET (Fig. 6b). In MAM and JJA, the relatively largest increase in ET mainly occurred in the middle of the study area, with the trend reaching up to 5 mm yr^−2^. However, some areas in the south parts, e.g., Ziwuling and Huanglong regions where productive forests are extensively distributed, ET decreased in JJA but increased slightly in MAM. In SON, such positive ET trend mainly occurred in the central and north regions of the LP, with ~88.8% of pixels being affected (Fig. 6c). By contrast, only some regions in the southeastern corner experienced obviously decreasing trend of the annual ET in DJF, which could be mainly ascribed to the significant decrease in the annual P (trend of 2 mm yr^−2^) (Fig. 6d).

### Vegetation greening and climate change controls on ET

**Figure 7:**
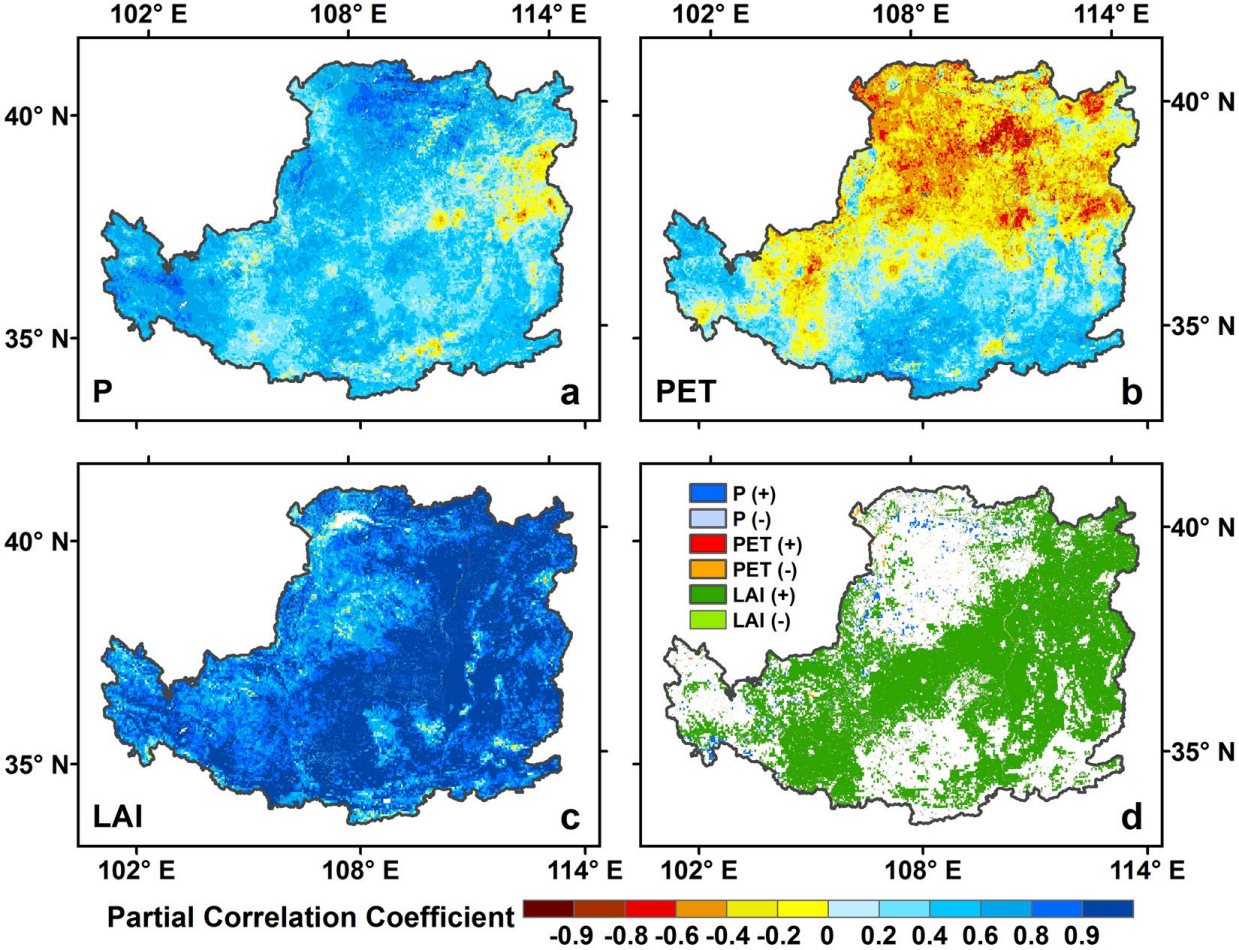
Spatial distribution of partial correlation coefficient (*r*) between annual ET and (**a**) P, (**b**) PET and (**c**) LAI, and (**d**) the dominant factors of the ET trend in the entire region in the LP from 2000 to 2012. Map was created using ArcMap 10.0 (http://www.esri.com/sofware/arcgis/arcgis-for-desktop).

Averaged the whole LP, despite the increase of annual ET over the study period, it showed large interannual fluctuations (Fig. 4a). Therefore, to investigate the climate and vegetation control on interannual variations of ET, we conducted partial correlation analysis between ET and P, PET and LAI for each grid over the LP during 2000–2012 (Fig. 7), with all variables detrended. The partial correlation coefficient (*r*) between the annual ET and LAI was significant at ∼90% of pixels (*p* < 0.05), and high *r* values were generally located in the strip region from the northeast to the southwest regions of the LP (Fig. 7c), where the LAI increased relatively rapidly (Fig. 5d). The *r* between the annual ET and P was also relatively higher in the whole study region, but only ~27.8% of pixels were significant (*p* < 0.05), which were generally distributed around the northern and western regions of LP where the annual mean P was relatively low (Fig. 2b). Compared with LAI and P, it could be seen that no coherent spatial patterns were shown in the relationship between the annual ET and annual PET with mixed positive and negative correlation in northern and southern parts of the LP respectively (~6.2% and ~3.8% with significant level, *p* < 0.05) (Fig. 7b).

Based on the spatial distribution patterns of *r*, the spatial distribution of dominant factors of ET changes in the entire region was shown in Fig. 7d (the *r* value in terms of the significant level between ET and climate/vegetation variables was higher than the sum of that from the other variables). The result showed that variations in LAI controlled the variability in ET at ~52.8% of pixels in the strip region from the northeastern to southwestern parts of the LP, where vegetation are greatly improved represented by a higher LAI increasing trend (Fig. 5d). On the contrary, variations in the annual ET over this region controlled by P and PET alone were only at ~1% of pixels, which were scattered in the northwestern parts of the LP. ET changes in the remainder of the study areas could be regarded as the combined effect resulting from climate and vegetation changes (~46%). Further analysis suggested that vegetation change is the major controlling factor on interannual variability of annual ET in all ecosystems (Fig. S3).

**Figure 8:**
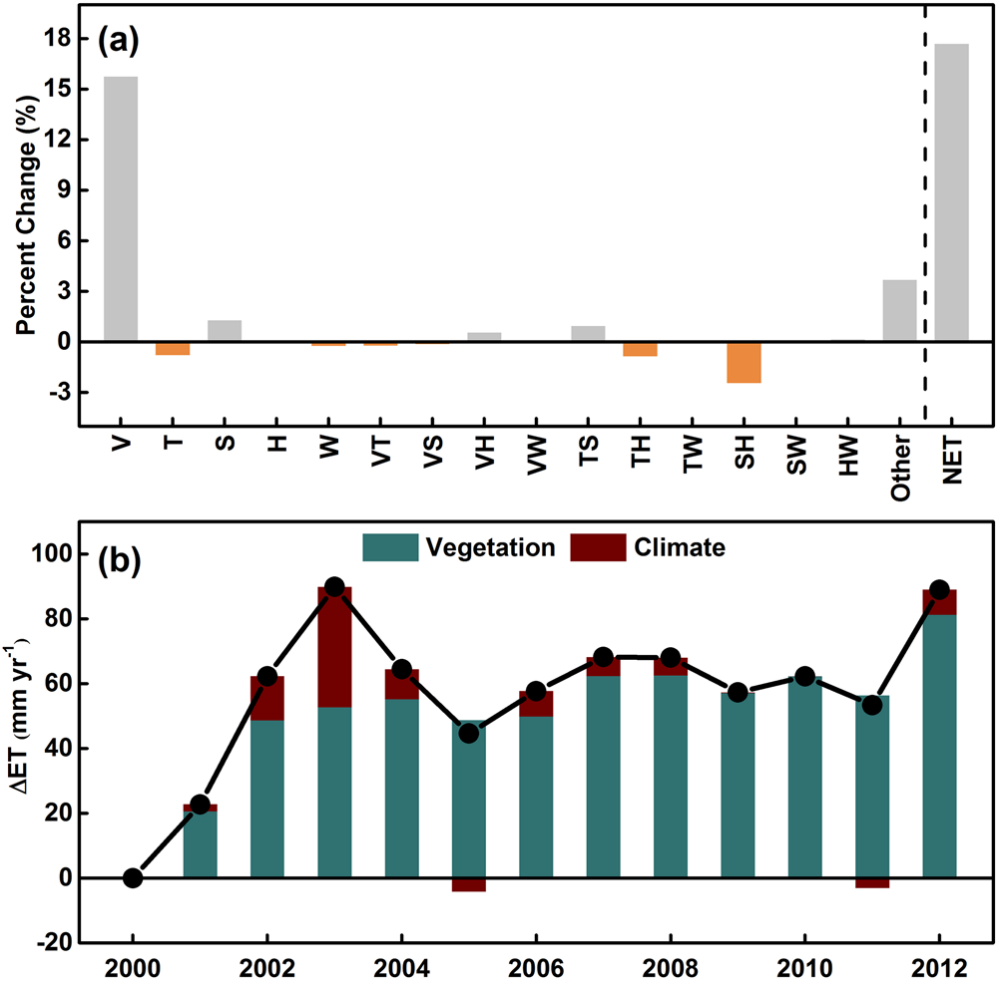
(**a**) Contributions of vegetation (V), air temperature (T), sunshine duration (S), relative humidity (R), wind speed (W), and their two-way and higher-order interactions affecting ET over the LP; (**b**) yearly contributions of V and sum of the other factors (climate change) affecting ET.

In order to examine the impacts of climate and vegetation change on ET trend in detail, PET were first separated into four major meteorological variables including mean air temperature (T), sunshine duration (S), relative humidity (H) and wind speed (W). Then we conducted the controlled factorial experiments under different scenarios (see Methods and also ref. 12). The model factorial simulation suggest that the average mean annual simulated net ET (all variables changing) was 17.7% higher than that of the control simulation (all variables fixed at the level of 2000), of which changes in LAI (15.7%), the two-way interactive effect of sunshine duration and vapor pressure (−2.4%), and the sum of all higher-order interactive effects (3.6%) explained the largest contribution to the decadal changes in ET of the LP (Fig. 8a). Changes in sunshine duration (1.2%), and two-way interactive effect of vegetation and vapor pressure (0.5%), interactive effect of sunshine duration and air temperature (0.9%) impose a slight positive effect on ET, whereas wind speed (−0.2%), air temperature (−0.7%), and interactive effect of air temperature and vapor pressure (−0.8%) have slight negative effects on ET. However, all these positive or negative effects are exceeded by the impact of LAI change (Fig. 8a), suggesting that vegetation greening is the primary driver of ET trend in the LP over the past thirteen years. Meanwhile, we found that the net effect of climate change is positive throughout the whole period except in 2005 (−4.1%) and 2011 (−3.0%), but much lower than the effect of vegetation change (Fig. 8b).

## Discussion

Climate change and anthropogenic disturbance are responsible for temporal and spatial change of ET^6, 41^. The controlling mechanisms that lead to differences in ET between regions indicate that studies on ET trends should be carried out at regional scales^42^. Gokmen, *et al*.^43^ suggested that trends of ET were mainly related to land cover changes and the intensification of the supplementary irrigation for agriculture in the Konya plain. Razyaseef, *et al*.^44^ concluded that with increasing tree transpiration and ecosystem water-use efficiency, the partitioning of ET appeared to be strongly influenced by temporal P patterns. Although the global land evapotranspiration trend showed a decline from 1998 to 2008^1^, the annual ET showed large interannual fluctuations with a significant increasing trend from 2000 to 2012 over the LP. Our results suggested that the variations in annual ET are attributed to the vegetation changes at more than half of the study areas. The ET upward trend is higher than that of P (3.35 mm yr^−2^ versus 2.04 mm yr^−2^), implying that agricultural production and food security will likely be threatened. Factorial simulation further suggest that vegetation greening promoted by the GFG project explained the largest contribution to the ET increasing trend of the LP, which is consistent with a recent work from a global terrestrial ET trends estimation^12^. Carbon dioxide (CO_2_) fertilization effects were the primary driving forces for observed greening trend (increase of LAI) over the Northern Hemisphere^45^, whereas some studied from process models suggested that China’s afforestation activity could better explain the spatial patterns of trend in vegetation growing^46^. This is particularly true in our study region. According to remote-sensed images, the average vegetation coverage in the LP has significantly increased to as much as 59.6% from the late 1990s to 2013^47^, instead of driving by elevated air temperature, changing precipitation, or rising atmospheric CO_2_ concentration^32^. The reasons why this large scale conversion of cropland to grasslands/forests or afforestation could cause ET increase are as follows.

First, at the biome level, forest ecosystems generally have a higher annual ET than that of herbage ecosystems on average due to higher total root biomass and deeper effective rooting depth^48^. Meanwhile, shrubs and forests may be featured by a longer active transpiration period than the herbaceous plant growth seasonally, thus leading to a higher total annual transpiration than that of croplands. With the tree growing, the increase of vegetation coverage might lead to more interception by canopy, and although the evaporation of intercepted precipitation in grasslands or croplands is generally low, it can account for 10–20% and 20–40% of rainfall for broadleaf trees and conifers respectively^49^. Second, afforestation can alter the surface energy exchange by affecting the land surface roughness, and give feedbacks to the regional ET^50^. The soil type in most areas of the LP is loessial soil with light color (i.e., yellow) while the vegetation has relatively darker color. And therefore, when the barren hills are afforested, the land surface albedo is expected to decline. Besides, forests generally have lower albedo than croplands and grasslands^50, 51^, suggesting that the conversion of croplands to forest can result in the decrease in albedo. This has also been proved by previous studies based on MODIS products, which indicated that the average albedo cross the LP significantly declined over the period 2000–2013 due to the GFG project^32^. All of these can result in an increase of the net shortwave radiation. On the assumption that all else being equal, there will be an increase in the upward longwave radiation (the longwave radiation emits from the surface into the atmosphere); however, this extra energy, resulting from lower albedo, can be dissipated with enhanced vegetation transpiration and soil evaporation^27^.

Contrary to the previous studies which stated that the LP has been experiencing a climatic warming and drying trend over the last several decades^52, 53^, air temperature have experienced a decreasing trend of −0.04 °C yr^−1^ during the recent decade even though it failed to pass the significance level test (*p* = 0.13, Fig. S4a). This observed cooling may result from vegetation biophysical feedbacks^54^. Consistent with a review that the recent slow-down in global near-surface winds^55^, wind speed over the plateau was also decreased (Fig. S4d), which is further attributed to increases in terrestrial surface roughness^56^. These above findings may explain that air temperature and wind speed exerted negative effects on the rises of ET over the LP (Fig. 8a). Meanwhile, we found that annual ET peaked in 2003, being 8% higher than the annual mean values (Fig. 4a). Further analysis showed that the high precipitation, and consequently relative humidity could explain this ET jump (Fig. S4c), despite a minor factor compared to LAI (2.11% versus 16.40%).

Although the vegetation index model have a good performance in predicting the annual ET over the LP, it is worthwhile to explore the uncertainties of the results. There is a limitation in this model in that it does not take into account of the impact of check-dam and irrigation on ET, thus resulting in a slight underestimation of ET (Fig. 1). Then again, some parameters used in the model were set as constant (the regression coefficients *a*_*s*_ and *b*_*s*_) or from a set of simple empirical formulation (albedo calculation), which will neglect the variability of model applicability at different time and space dimensions. In addition, the limited number and uneven distribution of the meteorological stations (e.g., few stations in the northern LP) can also affect the interpolation precision. Furthermore, different spatial resolution of data sources applied to drive the vegetation index model can be another cause of the discrepancies between the observed ET and the modeled ET. For example, the model simulated ET is at a 1-km scale, whereas LAI data are original 0.05 degree (about 5-km) resolution. Accordingly, vegetation index product with higher resolution should be employed in the future to enhance its ability to capture the spatial pattern of ET. In the ET validation, we used water balance ET (precipitation minus streamflow) which here constitutes the truth. Annual ET can be calculated as the residual between total precipitation and river discharge and terrestrial water storage change. However, we are not able to acquire such water storage data with high spatial resolution and accuracy for the entire study period. For example, despite the availability recently of terrestrial water storage datasets from gravity recovery and climate experiment (GRACE) satellites, the spatial resolution of this datasets is 1° (i.e., 10000 km^2^ for each grid), which is generally larger than the area of catchment used for validation. By contrast, the validation of our study was carried out on 13-year time scale (assumes hydrological steady state conditions) in which soil water storage was generally balanced even if vegetation was affected by human activities. Meanwhile, a recent study have reported that soil moisture decrease following the massive re-vegetation^57^. Therefore, if the soil moisture is considered in the water balance, the verification accuracy of the model will be more robust.

On the other hand, vegetation and climate have complex and interactive effects on terrestrial ET. Consequently, it is impossible to completely distinguish the effects of vegetation and climate change on ET as vegetation is also a response of the terrestrial ecosystems to climate change. For example, climate change (e.g., climate warming) could promote vegetation productivity through an extension in the growing season duration and enhanced nitrogen mineralization rate^58, 59^ and thus climate change could exert indirect effect on ET of the LP by influencing vegetation^12^. Despite the above weakness, our study do provide insight into the potential magnitude of vegetation and climate change impacts on ET. It is clear that both vegetation greening and climate change contributed the increase in ET over the LP (Fig. 8).

## Data and Method

### Study area and data

**Figure 9:**
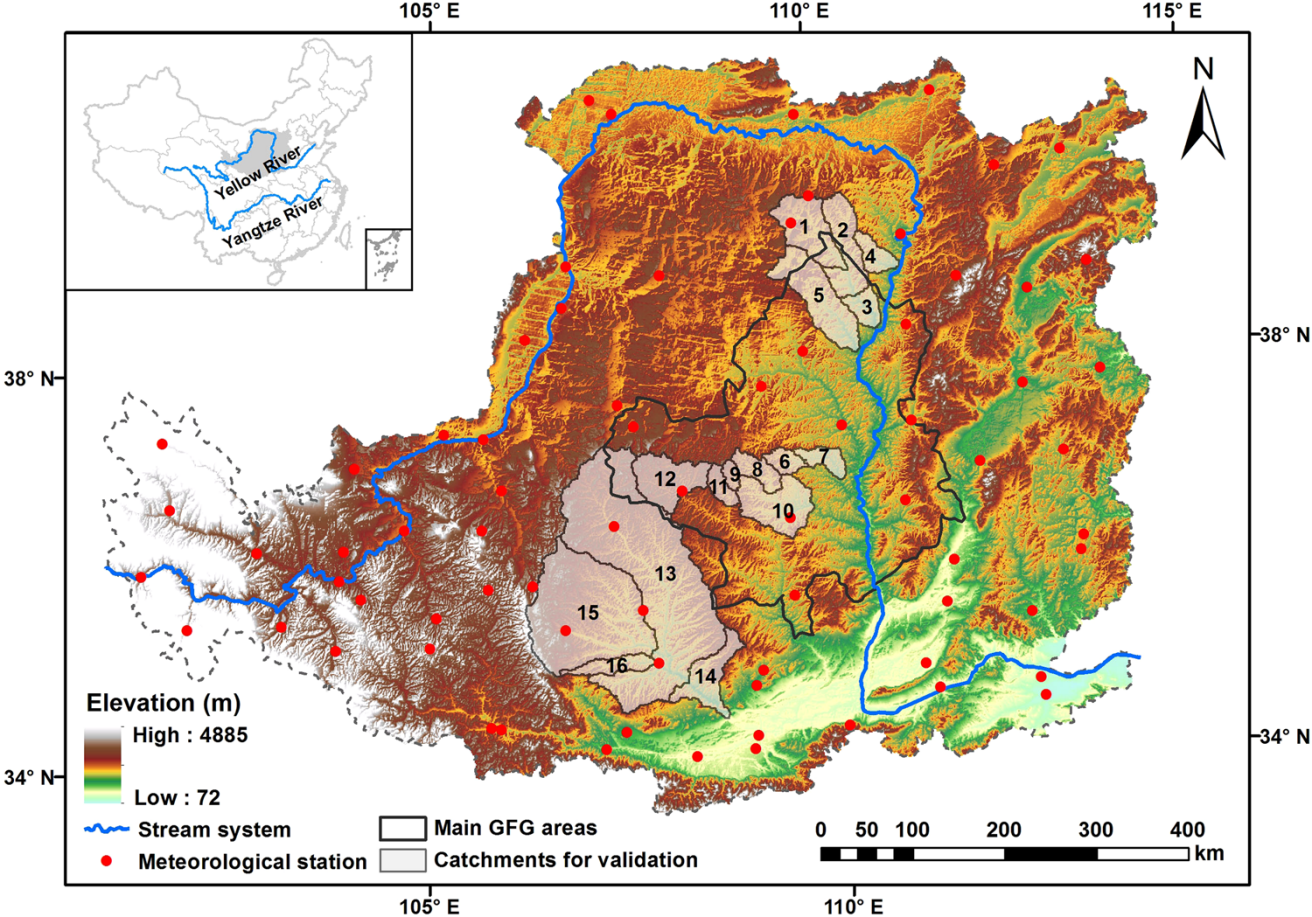
Study area of the Loess Plateau. The red dots are meteorological stations. The numbers on the catchments for validation correspond to the hydrological stations number provided in Table S1. Map was created using ArcMap 10.0 (http://www.esri.com/sofware/arcgis/arcgis-for-desktop).

The Loess Plateau (LP) of China, boasting a typical semi-arid and arid ecosystem, is located in the upper and middle reaches of the Yellow River (100°54–114°33E and 33°43–41°16N), and covers a total area of about 640,000 km^2^ (Fig. 9). The long-term (1960–2010) annual average precipitation is approximately 421 mm, of which 60–70% occurs between June and September in the form of high intensity rainstorms^60^. The annual average temperature is 9.0 °C with a minimum mean temperature of −4.6 °C in winter and a maximum annual temperature reaching to 20.9 °C in summer. Owing to the loose loess soils, steep slopes, periodic high intensity rainstorms and relatively sparse vegetation, the region is well-known for severe soil erosion and heavy sediment loads in the world. Besides, severe water shortages and limited natural conditions are also main obstacles for the sustainable development of this region^61^.

Meteorological forcing data including daily precipitation, air temperature, air pressure, relative humidity, wind speed and sunshine duration during the period 2000–2012, were obtained from China Meteorological Administration (CMA) at 65 stations located in and around the LP. These stations are well distributed in space and can reflect the characteristics of the regional climate. Most of these stations have collected complete data from 2000 to 2012. Missing daily data account for about 0.45% as an average for the 65 stations and were complemented by the median meteorological data from at least tree neighboring stations. All the meteorological data were spatially interpolated by the gradient inverse distance square (GIDS), which has been proven more precise compared with the ordinary Kriging and inverse distance squared interpolation method, as it could provide accurate estimates of spatial climatic variables by considering the effects of terrain, latitude and longitude through multivariate regressive analysis^62^. To further check the effects of meteorological stations’ density and the interpolation method on the climate variables interpolation in the LP, we randomly twice selected 80% and 90% of the original meteorological stations respectively, and spatially interpolated these data by the GIDS method. Results have showed that the GIDS interpolated data closely matched with the observed data (Supplementary Fig. S2), suggesting that this method have good performance on spatial interpolation of meteorological variables.

The Global Land Surface Satellite (GLASS) LAI data with a spatial resolution of 0.05 degree and a temporal resolution of 8 days from 2000 to 2012 were used for running the model (http://glass-product.bnu.edu.cn/). This product was retrieved from time-series Moderate Resolution Imaging Spectrometer (MODIS) and Advanced Very High Resolution Radiometer (AVHRR) surface reflectance data. Extensive validation for all biome types indicates that the GLASS LAI product can provide temporally-continuous LAI profiles with more improved quality and accuracy compared to the current MODIS and CYCLOPES products^62^. Therefore, the GLASS LAI product has been used widely for detecting the trend in vegetation, and can meet the needs of global change and climate studies^40^.

The black-sky albedo and the white-sky albedo were also obtained from GLASS dataset. The V1.0 GLASS albedo product provides a long-time series of high-quality, gap-filled, 8-day global 0.05° albedo map from 1981 to 2012^63^. The solar zenith angle (SEA) data was stemmed from the daily global 0.05° resolution surface reflectance datasets (https://www.ncdc.noaa.gov/cdr/terrestrial/avhrr-surface-reflectance) from AVHRR Long Term Data Record (LTDR). In order to unify the different time scales of multiple datasets and work out non-temporal data missing, we averaged daily SZA to obtain 8-daily data.

In order to characterize the land cover changes in the LP, we used the land cover maps in 2000, 2005, and 2010 with the resolution of 1 km for ET simulation. The maps were generated from the Institute of Geographic Sciences and Natural Resources Research, Chinese Academy of Sciences^64^. The China National Environmental Monitoring Center used historical U.S. Landsat Thematic Mapper/Enhanced Thematic Mapper (TM/ETM) images from 2000 to 2010 and the missing scenes were supplemented by adopting the data before or after these years. In addition, the land cover types were further differentiated for the required International Geosphere-Biosphere Program (IGBP) land cover classification system, including evergreen needleleaf, evergreen broadleaf, deciduous needleleaf, deciduous broadleaf, mixed forest, shrub, grassland, permanent wetlands, crop, urban, bare or sparse vegetation cover, and open water (Fig. S1).The datasets used in this study were further resampled to 1 km spatial resolution, and aggregated to annual average for January 2000 to December 2012.

Validation datasets include catchment streamflow and precipitation. In total, 15 largely unregulated catchments with a widespread geographic distribution were selected to assess model performance at the mean annual scale (Table S1). The annual streamflow data of the study period were obtained from the Yellow River Conservancy Commission (YRCC). By assuming that the annual change of water storage was negligible in the long term, the mean annual ET over the period 2000–2012 for each catchment was calculated as the mean annual precipitation minus streamflow. In addition, the annual MODIS ET product (MOD16) with a spatial resolution of 1 km from 2000 to 2011 were valid^65^. This product was derived from Numerical Terradynamic Simulation Group (NTSG; http://www.ntsg.umt.edu).

### Model description

The model adopts remotely sensed vegetation index and concurrent meteorological data as model inputs and estimates vegetation transpiration (*E*_c_) and soil evaporation separately (*E*_*s*_):

1 $${\rm{ET}}={E}_{c}+{E}_{s}$$

*E*_c_ is calculated as the potential transpiration rate (*E*_cp_) reduced by two scaling factors representing influences from air temperature and water stress:

2 $${E}_{c}={E}_{{\rm{cp}}}{f}_{w}{f}_{t}$$

where *f*_*w*_ and *f*_*t*_ are the stress functions of atmospheric water vapour pressure deficit and air temperature, respectively. Potential transpiration rate is calculated with the Penman-Monteith equation as:

3 $${E}_{cp}=\frac{1}{\lambda }({\Delta }{R}_{nc}+{f}_{c}\rho {C}_{p}D/{r}_{a})/({\Delta }+\gamma \eta )$$

where *λ* is the latent heat of vaporization of water (J kg^−1^); *Δ* is the slope of the curve relating saturated water vapour pressure to temperature (hPa °C^−1^); *R*_*nc*_ is the net radiation absorbed by canopy (MJ day^−1^); *f*_*c*_ is the fractional cover of vegetation; *ρ* is the air density (kg m^−3^); *C*_*p*_ is the specific heat capacity of air (J kg^−1^ °C^−1^); *D* is the saturated water vapour pressure deficit of air (hPa); *r*_*a*_ is the aerodynamic resistance between the canopy and the reference height (s m^−1^); *γ* is the latent heat of vaporization of water (J kg^−1^); *η* is the ratio of the minimum stomatal resistance (*r*_*s,min*_) of a natural plant functional type to that of the reference crop, and the minimum stomatal resistances are adopted as previous studies^25, 26^. *r*_*a*_ for short grassland is calculed as:

4 $${r}_{a}=\frac{{\mathrm{ln}}^{2}[(\,z-d)/{z}_{0}]}{{k}^{2}{u}_{a}}=\mathrm{208}/{u}_{a}$$

where *z* is the reference height; *d* is the zero plane displacement; *z*_0_ is the roughness length; *k* is the von Karman constant, 0.41; and *u*_*a*_ is the wind velocity. In this study, the differences for *d* and *z*_0_ between the natural vegetation types, which may cause insignificant bias in ET estimates are neglected in estimating *E*_*cp*_. The vegetation indices shows a good relationship with the root zone soil moisture deficit at large scales as most plants are capable of accessing soil water at depth^66^. Therefore, soil moisture could connect with regulate canopy transpiration through the change of *f*_*c*_. The expressions of constraints from air temperature (*f*_*t*_) and water vapour pressure deficit (*f*_*w*_) are given as^27, 28^:

5 $${f}_{t}=\exp (-{[({T}_{a}-{T}_{opt})/{T}_{opt}]}^{2})$$

6 $${f}_{w}=({\rm{D}}-{D}_{o})/({D}_{c}-{D}_{o})$$

where *T*_*a*_ is the air temperature; *T*_*opt*_ is the optimal temperature for canopy transpiration (20 °C); *D*_*o*_ and *D*_*c*_ are the water vapour pressure deficits when stomata starts to shrinking and closes completely (set as 6.5 and 38 hPa), respectively.

The intercepted evaporation from canopy is estimated as its potential rate over the wetted fraction of canopy. Soil evaporation (*E*_*s*_) is limited by the potential evaporation (*E*_*sp*_) and the soil exfiltration (*E*_*ex*_)^67^,

7 $${E}_{s}=\,{\rm{\min }}({E}_{sp},{E}_{ex})$$

8 $${E}_{{\rm{sp}}}=\frac{1}{\lambda }[{\Delta }({R}_{ns}-G)+({\rm{1}}-{f}_{c})\rho {C}_{p}D/{r}_{{\rm{as}}}]/({\Delta }+\gamma )$$

where *E*_*sp*_ is the potential evaporation rate (mm day^−1^); *R*_*ns*_ is the net radiation absorbed by soil surface (MJ day^−1^); *r*_*as*_ is aerodynamic resistance between reference height and soil surface; *G* is the soil heat flux (MJ day^−1^). *E*_*ex*_ (mm d^−1^) decreases with the depletion of surface soil moisture, expressed as ref. 68:

9 $${E}_{{\rm{ex}}}=S({t}^{{\rm{0.5}}}-{(t-{\rm{1}})}^{{\rm{0.5}}})$$

where *S* is the soil-controlled exfiltration volume, determined by the soil texture and structure, etc, which is usually falling by 3-mm d^−1.5^, and it is set a value of 2 mm d^−1.5^ in this study; *t* is the days, which elapsed since the day following rainfall.

The *f*_*c*_ is strongly related with remote sensing vegetation indices (VI), such as LAI (Leaf Area Index). The *f*_*c*_ is retrieved as follow^69^:

10 $${f}_{c}=1-{(\frac{{{\rm{VI}}}_{{\rm{\max }}}-{\rm{VI}}}{{{\rm{VI}}}_{{\rm{\max }}}-{{\rm{VI}}}_{{\rm{\min }}}})}^{\beta }$$

where *β* is empirical constant, being from 0.6 to 1.2, taken 0.8 here; VI_max_ and VI_min_ correspond to full vegetation cover and bare soil conditions, respectively.

The net radiation fluxes absorbed are divided with a weight of *f*_*c*_ for canopy and the soil surface underneath respectively, namely,

11 $${R}_{nc}={f}_{c}{R}_{n}$$

12 $${R}_{ns}={R}_{n}-{R}_{nc}$$

The calculation of net radiation (*R*_*n*_) using the following formula^70^:

13 $${R}_{n}={R}_{S}-{R}_{L}$$

14 $${R}_{S}=(1-\alpha ){R}_{solar}$$

15 $${R}_{solar}=({a}_{s}+{b}_{s}\frac{n}{N}){R}_{o}$$

16 $${R}_{L}=(0.1+0.9\frac{n}{N})(0.34-0.14\sqrt{{e}_{a}})\sigma {({T}_{a}+273)}^{4}$$

where *R*_*S*_ and *R*_*L*_ are the incoming net shortwave radiation and the outgoing net long-wave radiation (MJ d^−1^), respectively. *R*_solar_ is solar radiation, and it can be estimated from sunshine duration (or hours of sunshine) using the Angstrom-Prescott formula with the regression coefficients *a*_*s*_ and *b*_*s*_. Here, we used the averaged values the coefficients *a*_*s*_ (0.186) and *b*_*s*_ (0.556) at 10 stations near our study region with radiation observations obtained from Wang, *et al*.^16^. *α* is the land surface albedo; *n* and *N* are the actual and potential sunshine durations, respectively; *R*_*o*_ is the incoming solar radiation at the top of the atmosphere (MJ d^−1^); *e*_*a*_ is the air vapo pressure (hPa); and *σ* is the Stefan-Boltzmann constant.

We calculate the actual albedo (*α*) by combination of the black-sky albedo (*α*_*dir*_) and white-sky albedo (*α*_*dif*_), as follows:

17 $$\alpha ={f}_{dir}{\alpha }_{dir}+{f}_{dir}{\alpha }_{dif}$$

where *f*_*dir*_ and *f*_*dif*_ are the ratio of direct light and diffuse light in the total incoming light, respectively, and

18 $${f}_{dir}+{f}_{dif}=1.0$$

*f*_*dif*_ can be calculated from a simple power law equation of the form^71^:

19 $${f}_{dif}={a}^{\ast }{(\cos \theta )}^{b}$$

where *a* and *b* are regression coefficients (set as 0.123 and −0.842 in this study, respectively), *θ* is the solar zenith angle.

### Simulation strategy

In order to make the model simulation conform to the real scenarios for expressing the impacts of land cover change on the land surface evapotranspiration, we made the assumption that the land cover of the LP did not change for a certain period when land cover data were available. The land cover data for 2000, 2005, and 2010 were used for the periods of 2000–2004, 2005–2009, and 2010–2012, respectively.

In this study, to evaluate the contribution of each factor to the response variable, we compared the difference of modeled ET between the simulations with only one varied variable and control simulation^12, 71^. The control simulation is the simulation forced by the vegetation conditions (LAI, albedo and land cover) and meteorological variables at the level of 2000. The ET result from the control simulation indicates the value given the normal 2000’s vegetation and climate conditions. Meanwhile, according to previous studies^12, 72, 73^, we also calculated a two-way interaction between variable A and B which is the subtraction of the main effects of A and B from the effect of the joint A plus B treatment. The sum of all higher-order interactive effects (Other) is the difference between the effect of real condition and the sum of five main effects and ten two-way interactive effects. In total, we design 16 simulation scenarios (summarized in Table S2) to understand the effect of vegetation and climate change on annual ET over the LP from 2000 to 2012. More details about this factorial experiments could be found in ref. 12.

### Statistical analysis

The model performance in simulating ET used three criteria, i.e., root–mean –square-error (RMSE), the coefficient of determination (*R* ^2^) and mean bias (bias). A simple regression model was used to investigate trends in annual ET and other variables (P, PET and LAI) for the period 2000–2012. The partial correlation coefficient (*r*) was calculated to show the strength of the ET-climate and the ET-LAI relations.

## Electronic supplementary material

Supplementary Information
