# Supplementary material for: Separating Vegetation Greening and Climate Change Controls on Evapotranspiration trend over the Loess Plateau
Source: Sci Rep. 2017 Aug 15;7:8191. doi: 10.1038/s41598-017-08477-x (PMC5557839; doi:10.1038/s41598-017-08477-x)
Supplement: Supplementary file 1 — Supplementary Information [file 41598_2017_8477_MOESM1_ESM.doc]

Title: Separating Vegetation Greening and Climate Change Controls on Evapotranspiration trend over the Loess Plateau

**List of authors:**

Zhao Jin^1,2^, Wei Liang^1,2,3,4^, Yuting Yang^5^, Weibin Zhang^1,2^, Jianwu Yan^1,4*^, Xuejuan Chen^6^, Sha Li^1^, Xingguo Mo^6^

1. School of Geography and Tourism, Shaanxi Normal University, Xi’an 710119, China

2. Shaanxi Key Laboratory of Tourism Informatics, Xi’an 710119, China

3. State Key Laboratory of Urban and Regional Ecology, Research Center for Eco-Environmental Sciences, Chinese Academy of Sciences, Beijing 100085, China

4. National Demonstration Center for Experimental Geography Education, Shaanxi Normal University, Xi’an 710119, China

5. CSIRO Land and Water, Canberra, ACT 2601, Australia

6. Key Laboratory of Water Cycle and Related Land Surface Processes, Institute of Geographical Sciences and Natural Resources Research, Chinese Academy of Sciences, Beijing 100101, China

**Corresponding Authors Contact Details:**

Email: yanjw@snnu.edu.cn;

Tel (Fax): +86 (29) 85310525;

Address: College of Tourism and Environment, Shaanxi Normal University, Xi’an 710119, China

**Article type:** Primary Research Article

**Supplementary Table S1** Long-term mean annual (2000-2011) precipitation (P) and streamflow (Q) of the catchments in the Loess Plateau used in this study.

| Catchment | Gauge Station | Station ID | Area (km^2^) | Longitude | Latitude (N) | Annual P (mm yr^-1^) | Annual Q |
| --- | --- | --- | --- | --- | --- | --- | --- |
|  |  |  |  | (E) |  |  | (mm yr^-1^) |
| Kuye River | Wangdaohengta | 1 | 3839 | 110°24′ | 39°04′ | 359.5 | 17 |
|  | Shenmu | 2 | 7298 | 110°30′ | 38°48′ | 368.6 | 18.8 |
|  | Wenjiachuan | 3 | 8515 | 110°45′ | 38°29 ′ | 373.6 | 18.8 |
| Tuwei River | Gaojiachuan | 4 | 3253 | 110°29′ | 38°15′ | 383.6 | 64.7 |
| Gushanchuan | Gaoshiya | 5 | 1263 | 111°03′ | 39°03′ | 384.6 | 13.1 |
| Qingjian River | Zichang | 6 | 913 | 109°42′ | 37°09′ | 451.5 | 36.3 |
|  | Yanchuan | 7 | 3468 | 110°11′ | 36°58′ | 452.6 | 28.7 |
| Yan River | Ansai | 8 | 1334 | 109°19′ | 36°52′ | 445.3 | 34.2 |
|  | Xinghe | 9 | 479 | 108°52′ | 36°56′ | 439.7 | 31.2 |
|  | Ganguyi | 10 | 5891 | 109°48′ | 36°42′ | 464 | 24.4 |
| Beiluo River | Zhidan | 11 | 774 | 108°46′ | 36°49′ | 443.3 | 20.6 |
|  | Wuqi | 12 | 3408 | 108°12′ | 36°53′ | 409.3 | 18.1 |
| Jing River | Jingcun | 13 | 40281 | 108°08′ | 35°00′ | 480.2 | 24.2 |
|  | Zhangjiashan | 14 | 43216 | 108°36′ | 34°38′ | 486.7 | 23.3 |
|  | Yangjiaping | 15 | 14124 | 107°4′ | 35°20′ | 486.8 | 29.7 |
|  | Zhanghe | 16 | 1506 | 107°43′ | 35°11′ | 546.7 | 38.1 |

**Supplementary Table S2** Design of Simulation Scenarios in This Study. Y: the input variable changes from 2000 to 2012, and N: the input variable is fixed as the level in 2000.

| **Scenario** | **S** | **T** | **W** | **H** | **V** |
| --- | --- | --- | --- | --- | --- |
| *f*(control) | N | N | N | N | N |
| *f*(S) | Y | N | N | N | N |
| *f*(T) | N | Y | N | N | N |
| *f*(H) | N | N | N | Y | N |
| *f*(W) | N | N | Y | N | N |
| *f*(S,T) | Y | Y | N | N | N |
| *f*(S,H) | Y | N | N | Y | N |
| *f*(S,W) | Y | N | Y | N | N |
| *f*(T,H) | N | Y | N | Y | N |
| *f*(T,W) | N | Y | Y | N | N |
| *f*(H,W) | N | Y | N | Y | N |
| *f*(V) | N | N | N | N | Y |
| *f*(S,V) | Y | N | N | N | Y |
| *f*(T,V) | N | Y | N | N | Y |
| *f*(H,V) | N | N | N | Y | Y |
| *f*(W,V) | N | N | Y | N | Y |

**
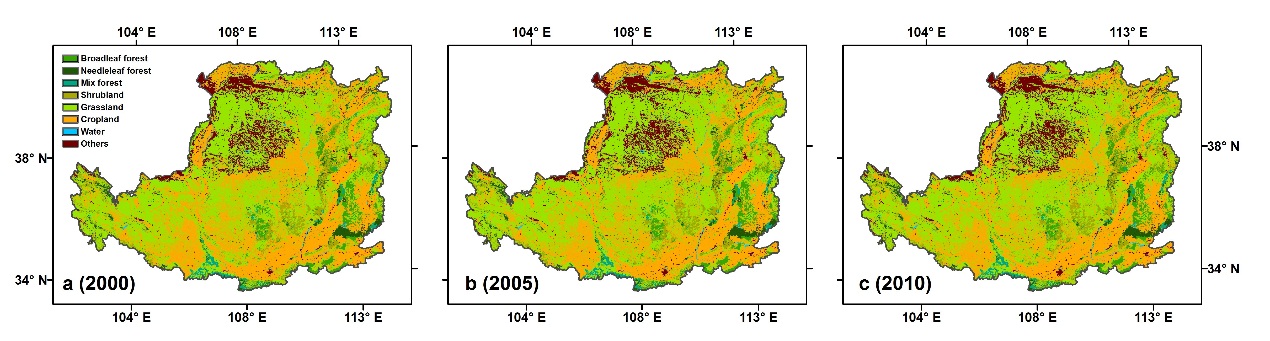
**

**Supplementary Figure S1** Distribution of land cover types in the Loess Plateau in (a) 2000, (b) 2005, and (c) 2010. Map was created using ArcMap 10.0 (<http://www.esri.com/sofware/arcgis/arcgis-for-desktop>).


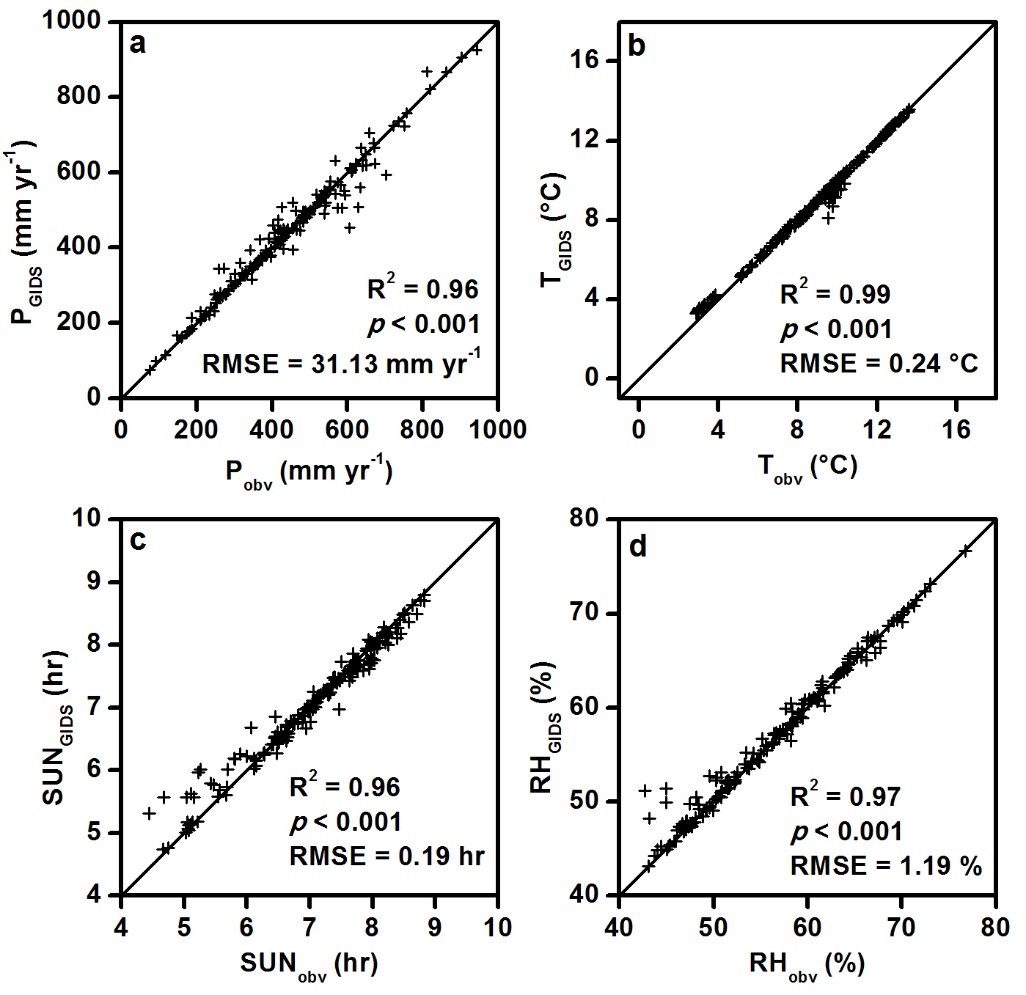


**Supplementary Figure S2(a)** We randomly selected 90% of the original meteorological stations for two times, respectively, and spatially interpolated these data (i.e., [a] precipitation, [b] mean temperature, [c] sunshine duration and [d] relative humidity), by gradient inverse distance square (GIDS) method. Climate variables for the remaining site were further extracted, and we made a comparison between these extracted data and observed ones.


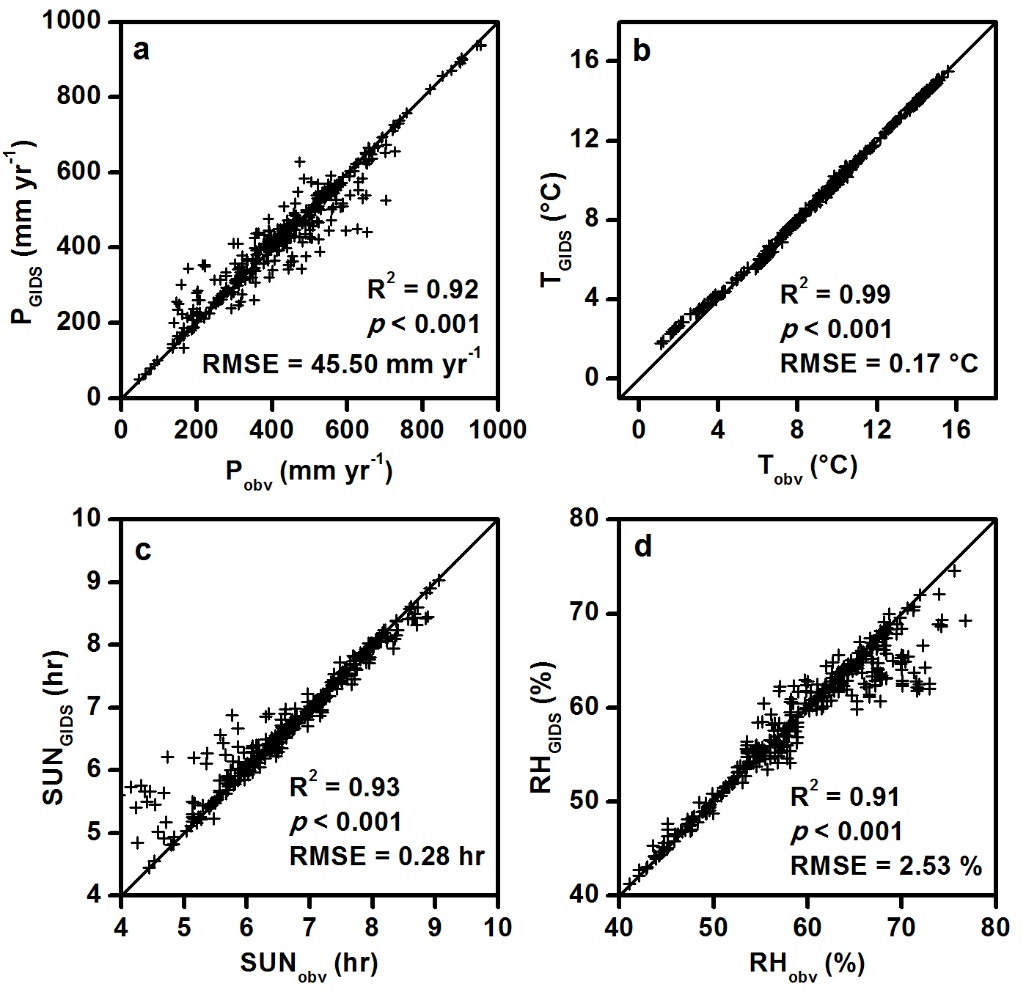


**Supplementary Figure S2 (b)** Same as in Figure S2(a), but for 80% of the original meteorological stations.


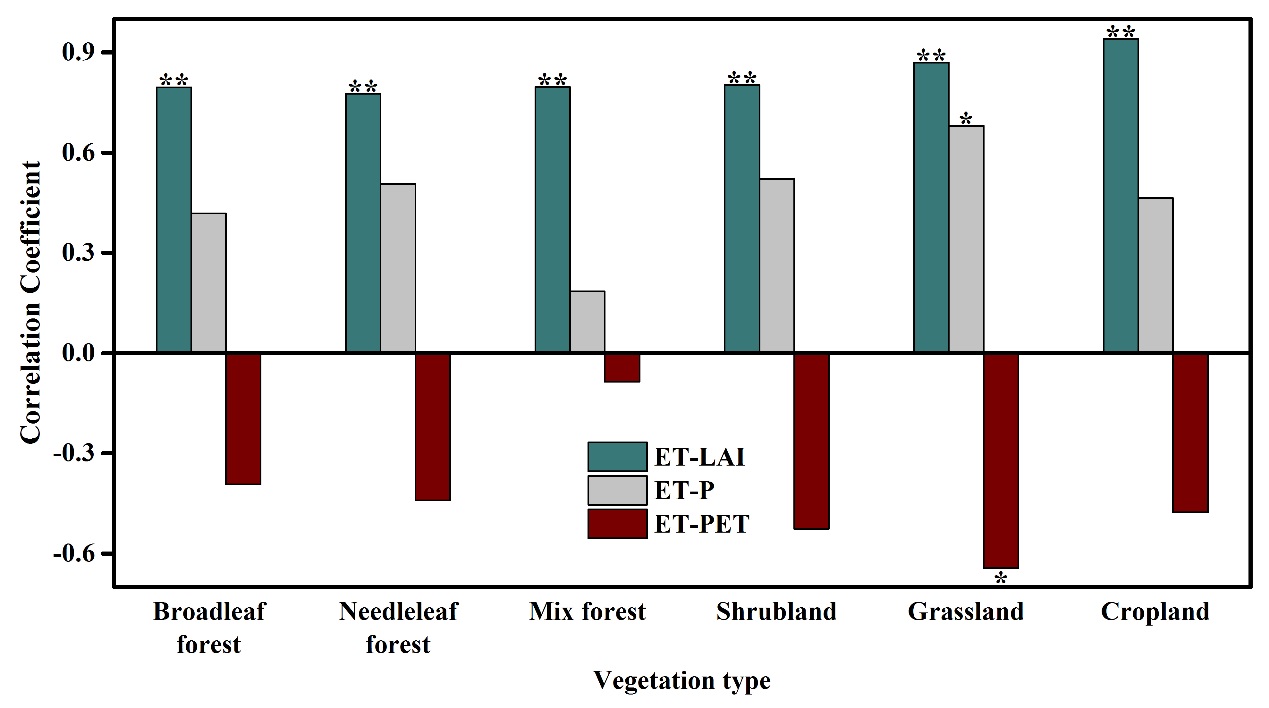


**Supplementary Figure S3** Correlation coefficient between ET and LAI, P, and PET at the annual scale for each vegetation type, including broadleaf forest, needleleaf forest, mix forest, shrubland, grassland, and cropland. ***p* <0.01; **p* <0.05.


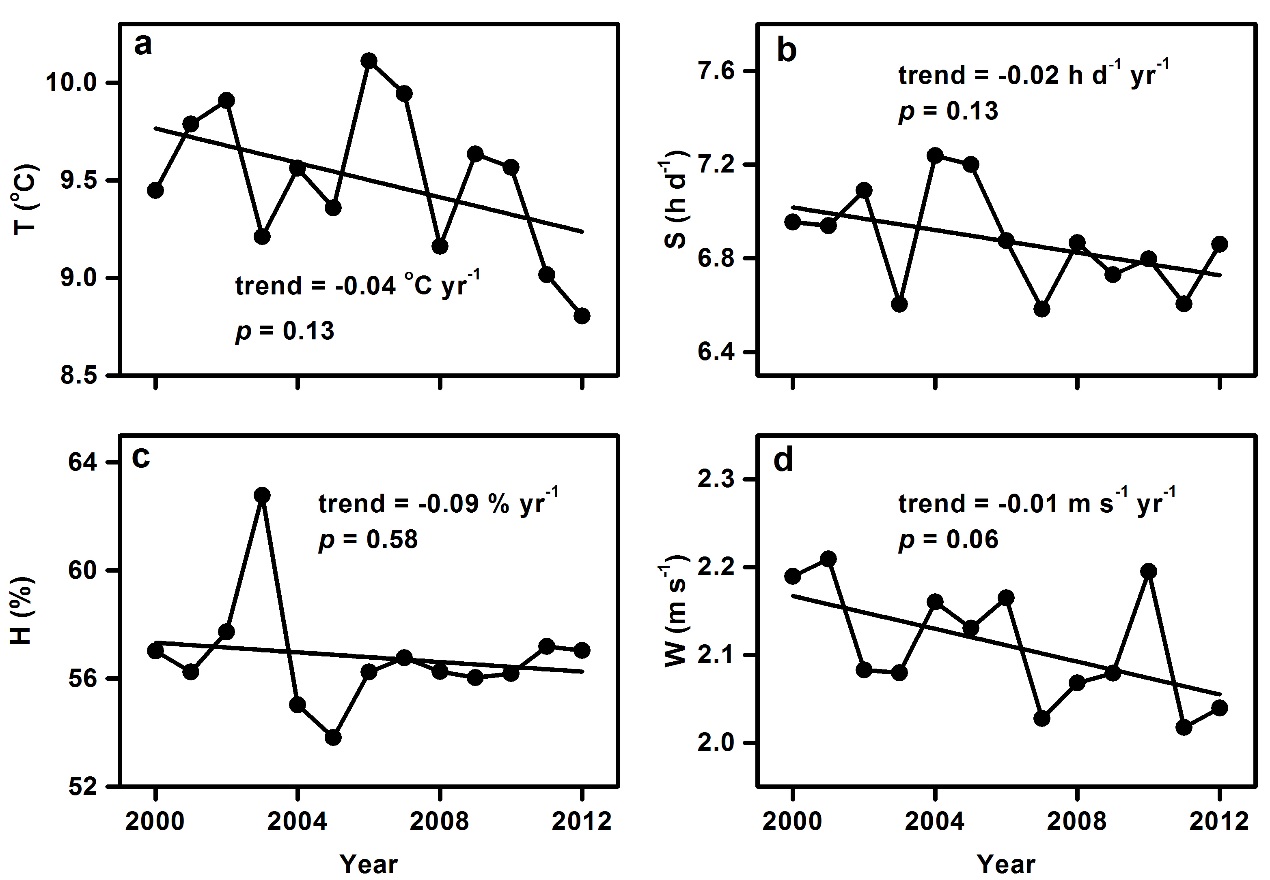


**Supplementary Figure S4** Interannual variations in mean (a) air temperature (T), (b) sunshine duration (S), (c) relative humidity (H) and (d) wind speed (W) over the LP from 2000 to 2012 (black dots). Solid black lines show the best fit linear relationship for the whole period.
